# Subtypes of hypertension and their association to comorbidities and ethnicity in pregnant women

**DOI:** 10.1371/journal.pone.0326649

**Published:** 2025-06-25

**Authors:** Daniel Perejón López, Nancy Melissa Medrano Duarte, María Catalina Serna Arnáiz, Miriam Orós Ruiz, Júlia Siscart Viladegut, Iñaki Gascó Serna

**Affiliations:** 1 Primary Care Research Institute IDIAP Jordi Gol, Catalan Institute of Health, Lleida, Spain; 2 Eixample Health Center, Catalan Institute of Health, Lleida, Spain; 3 Family Medicine Department, University of Lleida, Lleida, Spain; 4 Serós Health Center, Catalan Institute of Health, Lleida, Spain; 5 University Hospital Germans Trias i Pujol, Barcelona, Spain; Rift Valley University, ETHIOPIA

## Abstract

**Objectives:**

We analyzed the subtypes of arterial hypertension and their relationship with different comorbidities and ethnicity during pregnancy.

**Study design:**

This is a retrospective observational cohort study in 17,177 pregnant women during the years 2012–2018 in the health region of Lleida.

**Main outcome measures:**

We analyzed the relationship of chronic hypertension, gestational hypertension, and pre-eclampsia with different variables, including comorbidities, vascular risk factors, and ethnicity. We calculated the adjusted odds ratio (OR) and the 95% confidence interval (CI) with multivariate logistic regression models.

**Results:**

The total prevalence of arterial hypertension among pregnant women was 3.10%; of these, 1.53% had chronic hypertension, 0.65% had gestational hypertension, and 0.78% had pre-eclampsia. Superimposed pre-eclampsia on chronic hypertension occurred in 25 cases (0.14%). Chronic hypertension was associated with age (OR = 1.07), overweight (OR = 4.32), obesity (OR = 2.11), diabetes mellitus (OR = 2.38), hypothyroidism (OR = 2.23), and sub-Saharan origin (OR = 3.36). Gestational hypertension was correlated with age (OR = 1.06), overweight (OR = 5.84), obesity (OR = 1.89), and Asian/Middle Eastern origin (OR = 3.71). Finally, pre-eclampsia was associated with overweight (OR = 2.37) and Sub-Saharan Africa origin (OR = 2.45).

**Conclusions:**

Pregnant women with hypertension may benefit from the knowledge of the subtype and a consequent coordinated prenatal and pregnancy approach.

## Introduction

Hypertension during pregnancy is one of the leading causes of maternal and fetal morbidity and mortality worldwide and is responsible for approximately 14% of maternal deaths globally [1,2]. Specifically, hypertension in pregnancy is associated with an increased risk of placental abruption, intrauterine growth restriction, preterm delivery, renal failure, pre- and post-partum hemorrhage, and perinatal and maternal death [3–6].

According to the International Society for the Study of Hypertension in Pregnancy, arterial hypertensive disorders in pregnancy were classified as follows: chronic hypertension, detected before pregnancy or before 20 weeks’ gestation; gestational hypertension, which occurs de novo from 20 weeks’ gestation onwards; and pre-eclampsia, namely gestational hypertension accompanied by new-onset proteinuria, maternal organ dysfunction, or evidence of uteroplacental dysfunction. Also, pre-eclampsia can superimpose on chronic hypertension [7].

Numerous studies have explored the prevalence and risk factors of preeclampsia [8,9]; however, there is limited data concerning chronic hypertension and gestational hypertension. Thus, we aimed to investigate the associations between various subtypes of hypertension during pregnancy and comorbidities, vascular risk factors, and the ethnic origin of pregnant women.

## Methods

### Design and data collection

This is a retrospective observational cohort study in pregnant women in the health region of Lleida, at the University Hospital Arnau de Vilanova, between 2012 and 2018.

Data were obtained from the basic minimum dataset (CMBD: “conjunto mínimo de la base de datos”) of the electronic medical records database e-CAP, and electronic prescriptions of the Catalan Health Service.

This study is part of the ILERPREGNANT project. The main objective of ILERPREGNANT is to analyze the prevalence of different conditions, therapeutic prescription, and pharmacological adherence during pregnancy [10].

### Participants

Women who had given birth between 1 January 2012 and 31 December 2018 were included in the study. Pregnancy data were collected from the date of the last menstrual period to the date of delivery. Accordingly, information from 2011 was considered for women with a delivery date in 2012 whose last menstrual period was in 2011. To assess the representativeness of the sample, we calculated the percentage of births studied (those registered at the University Hospital Arnau de Vilanova in Lleida) in relation to the total number of births in the health region of Lleida (data obtained from the database of the Statistical Institute of Catalonia (Idescat) (Table 1).

**Table 1:** Number of deliveries registered in the health region of Lleida by years as per Idescat, and number of deliveries of the sample studied and percentages they represent.

Year	Deliveries (Idescat)	Deliveries (sample)	Idescat/sample
**2012**	3788	3635	96%
**2013**	3535	3370	95%
**2014**	3592	3308	92%
**2015**	3426	3162	92%
**2016**	3283	3180	97%
**2017**	3197	3034	95%
**2018**	3029	3001	99%

### Variables measured

The primary variable was the diagnosis of hypertensive disorder in pregnancy according to ICD-10-CM classification: chronic hypertension (I10) (≥140/90 mmHg (before 20 weeks)), gestational hypertension (O13.9)(≥140/90 mmHg (after 20 weeks, without proteinuria)), pre-eclampsia (O14.90) (140–159/90–109 mmHg with proteinuria), and superimposed pre-eclampsia on chronic hypertension (O11.09) (≥140/90 mmHg with proteinuria (after 20 weeks)). Secondary maternal variables included the following: age; ethnicity; body mass index (BMI) (Z68); number of gestations; gestational age; type of delivery; maternal history of overweight (E66.1); obesity (E66.9); diabetes mellitus (E11.9); gestational diabetes (O24.419); dyslipidemia (E78.9); depression (F32.9)); hypothyroidism (E03.9); multiple pregnancy.

### Data analysis

We performed a descriptive analysis representing numerical variables with mean and standard deviation, and categorical variables with absolute and relative frequencies. Group differences were evaluated using the Student’s t-test for numerical variables or Chi-square test for categorical variables.

We examined the association of the variables with the prevalence of the different types of hypertension through a multivariate linear model. The latter was constructed by purposefully selecting variables on the basis of clinical relevance, with prevalence of hypertension as the response variable and the remaining ones as predictors. We calculated regression coefficients, 95% confidence intervals, and p-values for chronic, gestational hypertension and pre-eclampsia.

### Ethics

This study was approved by the Clinical Research Ethics Committee (CREC) of the research institute *IDIAP Jordi Gol* (code 19/195-P) and adheres to the Declaration of Helsinki. It follows the tenets of the Declaration of Helsinki. The information was extracted from centralized medical files in the e-CAP database by the Health Research and Assessment Management Department. Informed consent from participants was not needed. The variables in the e-CAP database were processed anonymously and with all the guarantees of confidentiality established by the National Law and Regulation 2016/679 of the European Parliament and Council on the protection of individuals with regard to the use of personal information.

## Results

**Fig 1:**
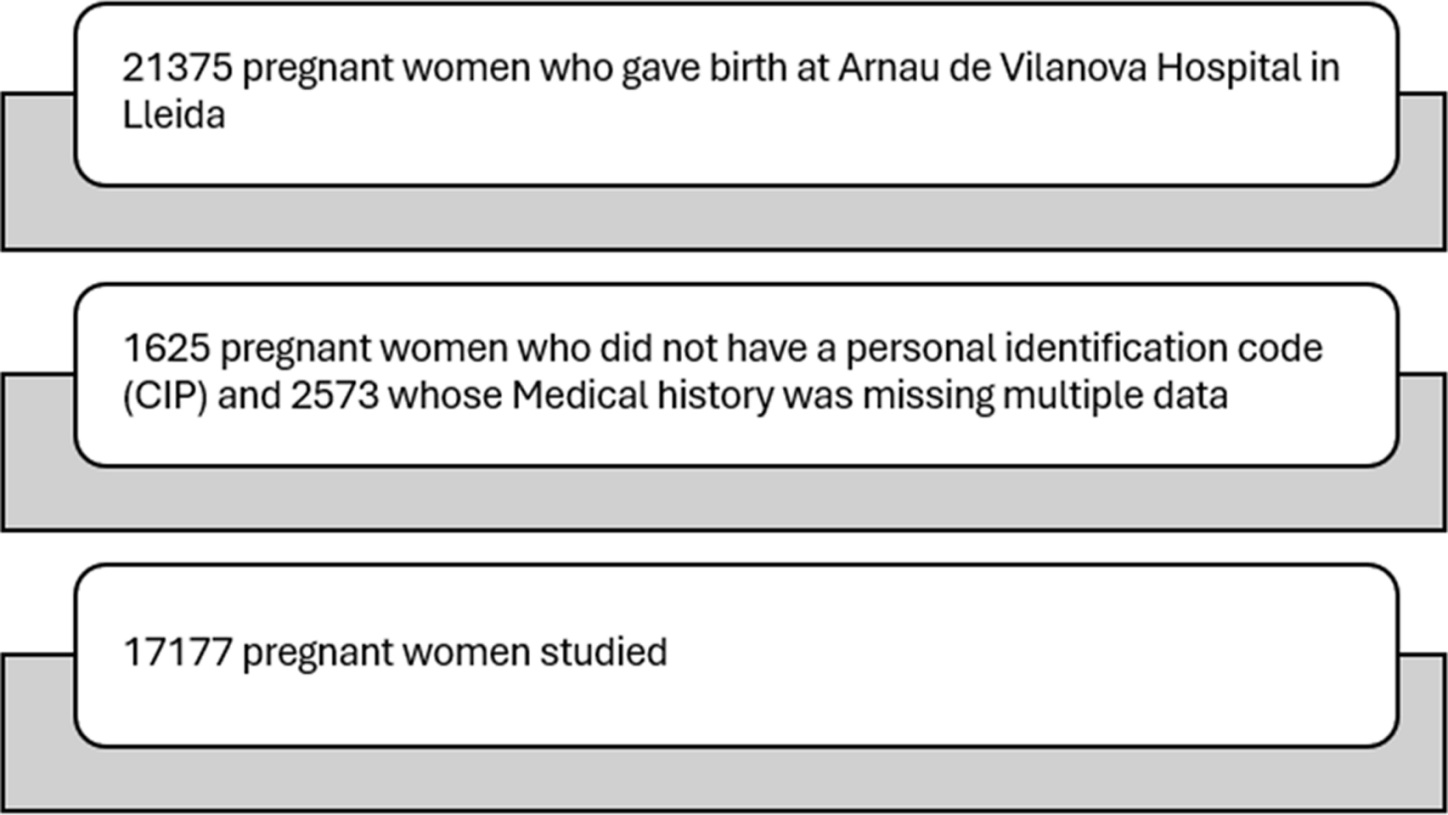
Sample of pregnant women included in the study.

A total of 21,375 women gave birth at the University Hospital Arnau de Vilanova in Lleida, between 2012 and 2018 (inclusive). Of these, we excluded 1,625 because of absence of a personal identification code (PIC) and 2,573 for other reasons, including loss of data on clinical history. Consequently, we analyzed 17,177 pregnant women (Fig 1), of which 533 (3.10%) had the diagnosis of arterial hypertension. Specifically, 263 (1.53%) had chronic hypertension, 111 (0.65%) were diagnosed with gestational hypertension, and 134 (0.78%) with pre-eclampsia. Additionally, superimposed pre-eclampsia on chronic hypertension occurred in 25 cases (0.14%). Of the total number of pregnant women with chronic hypertension, 87 (33.1%) were in treatment.

We explored the association between hypertension and different variables within the study population (Table 2). Notably, women with chronic hypertension had a lower mean age (33.9 ± 6.2 years) than normotensive women (30.6 ± 5.8).

**Table 2:** Characteristics of patients stratified according to subgroups of hypertension during pregnancy.

	Chronic Hypertension	Gestational Hypertension	Pre-eclampsia	Non-hypertension	p-value overall
	*n = 263*	*n = 111*	*n = 134*	*N = 16644*	
**Year of delivery:**					
**2011**	0 (0.00%)	0 (0.00%)	0 (0.00%)	43 (100%)	
**2012**	32 (1.17%)	16 (0.59%)	19 (0.69%)	2668 (97.55%)	
**2013**	37 (1.47%)	18 (0.71%)	17 (0.67%)	2451 (97.15%)	
**2014**	31 (1.25%)	9 (0.36%)	22 (0.89%)	2423 (97.50%)	
**2015**	50 (2.07%)	11 (0.46%)	23 (0.95%)	2331 (96.52%)	
**2016**	31 (1.28%)	16 (0.66%)	30 (1.24%)	2338 (96.81%)	
**2017**	46 (1.99%)	22 (0.95%)	15 (0.65%)	2231 (96.41%)	
**2018**	36 (1.62%)	19 (0.86%)	8 (0.36%)	2159 (97.16%)	
**Age of the patients**	33.9 (6.2)	32.7 (5.5)	30.6 (5.5)	30.6 (5.8)	<0.001
**BMI**	28.8 (6.2)	29.3 (6.4)	25.9 (5.8)	24.8 (4.9)	<0.001
**BMI (qualitative):**					
**Underweight**	4 (0.65%)	2 (0.33%)	3 (0.49%)	602 (98.5%)	
**Normal weight**	70 (0.75%)	31 (0.33%)	66 (0.71%)	9184 (98.2%)	
**Obesity**	98 (3.99%)	49 (2.00%)	26 (1.06%)	2281 (93.0%)	
**Overweight**	83 (1.90%)	29 (0.66%)	34 (0.78%)	4216 (96.7%)	
**Pregnancy number:**					
**1**	108 (1.20%)	57 (0.63%)	94 (1.04%)	8737 (97.1%)	
**2**	82 (1.59%)	32 (0.62%)	28(0.54%)	5030 (97.3%)	
**3**	39 (2.09%)	16(0.86%)	7 (0.37%)	1808 (96.7%)	
**4**	20 (3.11%)	5 (0.78%)	3(0.47%)	616 (95.7%)	
**>4**	14 (2.98%)	1 (0.21%)	2(0.43%)	453 (96.4%)	
**Multiple pregnancy:**					1.000
**No**	263 (1.54%)	111 (0.65%)	134 (0.78%)	16612 (97.0%)	
**Yes**	0 (0.00%)	0 (0.00%)	0 (0.00%)	32 (100.0%)	
**Diagnosis of hypothyroidism:**					0.002
**No**	230 (1.43%)	103 (0.64%)	126 (0.79%)	15569 (97.1%)	
**Yes**	33 (2.94%)	8 (0.71%)	8 (0.71%)	1075 (95.6%)	
**Diagnosis of hypothyroidism during pregnancy:**					0.435
**No**	260(1.53%)	109(0.64%)	132 (0.78%)	16481 (97.0%)	
**Yes**	3 (1.76%)	2 (1.18%)	2 (1.18%)	163 (95.9%)	
**Diagnosis of gestational hypertension:**					<0.001
**No**	263 (1.54%)	0 (0.00%)	134(0.79%)	16644 (97.7%)	
**Yes**	0 (0.00%)	111 (100%)	0 (0.00%)	0 (0.00%)	
**Diagnosis of diabetes mellitus:**					<0.001
**No**	203 (1.29%)	95 (0.60%)	120 (0.76%)	15332 (97.3%)	
**Yes**	60 (4.28%)	16 (1.14%)	14 (1.00%)	1312 (93.6%)	
**Diagnosis of gestational diabetes mellitus:**					<0.001
**No**	223 (1.39%)	98 (0.61%)	124 (0.77%)	15575 (97.2%)	
**Yes**	40 (3.53%)	13 (1.15%)	10(0.88%)	1069 (94.4%)	
**Dyslipidemia diagnosis:**					<0.001
**No**	250 (1.47%)	108 (0.64%)	132 (0.78%)	16475 (97.1%)	
**Yes**	13 (6.95%)	3 (1.60%)	2 (1.07%)	169 (90.4%)	
**Depression diagnosis:**					0.648
**No**	257 (1.54%)	106(0.63%)	130(0.78%)	16224 (97.1%)	
**Yes**	6 (1.38%)	5 (1.15%)	4 (0.92%)	420 (96.6%)	
**Depression in treatment:**					1.000
**No**	262 (1.53%)	111 (0.65%)	134 (0.78%)	16579 (97.0%)	
**Yes**	1 (1.52%)	0 (0.00%)	0 (0.00%)	65 (98.5%)	
**Region:**					
**Sub-Saharan Africa**	44 (5.26%)	12 (1.43%)	17 (2.03%)	764 (91.3%)	
**Latin America**	12 (1.68%)	7 (0.98%)	7 (0.98%)	690 (96.4%)	
**Asia and the Middle** **East**	3 (1.35%)	4 (1.80%)	1 (0.45%)	214 (96.4%)	
**Europe**	122 (1.29%)	56 (0.59%)	68 (0.72%)	9200 (97.4%)	
**Eastern Europe**	17 (1.11%)	8 (0.52%)	15 (0.98%)	1491 (97.4%)	
**Maghreb**	23 (1.03%)	19 (0.85%)	11 (0.49%)	2177 (97.6%)	

Analyzing the BMI, 37.3% of women with chronic hypertension, 44.1% of women with gestational hypertension, and 19.4% of women with pre-eclampsia were obese at the beginning of pregnancy, in comparison with 14.0% of normotensive women.

Diabetes mellitus was present in 22.8% of chronic hypertensive women, 14.4% of women with gestational hypertension, and 10.4% of the ones with pre-eclampsia, in comparison with 7.9% of normotensive pregnant women.

The prevalence of hypothyroidism was higher in pregnant women with chronic hypertension (12.5%), in comparison with normotensive ones (6.5%). For women with gestational hypertension and pre-eclampsia, it was alike normotensive ones (7.2% and 6.0% respectively).

Dyslipidemia was much higher in women with chronic hypertension (4.9%), women with gestational hypertension (2.7%), and women with pre-eclampsia (1.5%), than in normotensive ones (1.0%).

Examining the data by ethnic origin, Sub-Saharan Africa women showed the highest prevalence of chronic hypertension (5.26%) and pre-eclampsia (2.03%). Conversely, the Maghreb population showed the lowest prevalence of chronic hypertension (1.03%).

**Fig 2:**
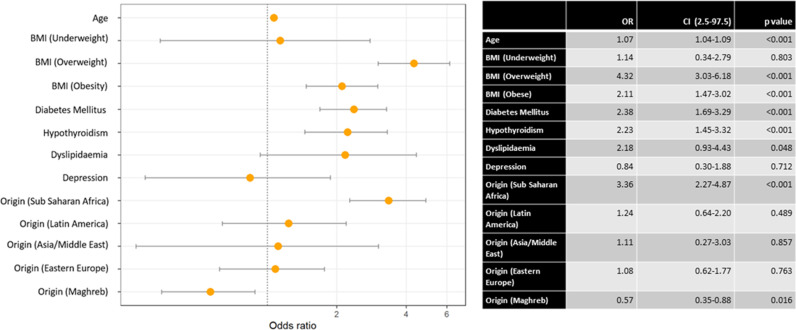
Multivariate analysis of chronic hypertension with various comorbidities, vascular risk factors and ethnicity.

**Fig 3:**
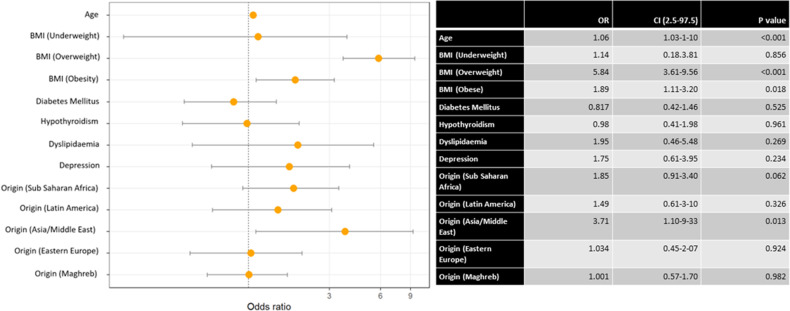
Multivariate analysis of gestational hypertension with various comorbidities, vascular risk factors and ethnicity.

**Fig 4:**
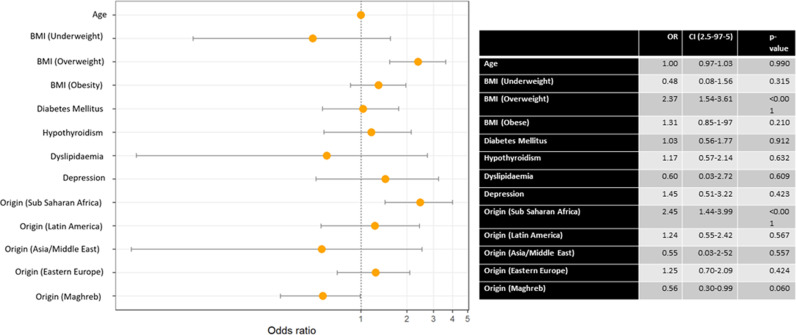
Multivariate analysis of pre-eclampsia with various comorbidities, vascular risk factors and ethnicity.

We performed a multivariate analysis on the different variables for chronic hypertension (Fig 2), gestational hypertension (Fig 3) and pre-eclampsia (Fig 4).

We found a positive association between age and chronic hypertension (OR = 1.07).

Being overweight showed a strong positive association with chronic hypertension (OR = 4.32) and pre-eclampsia (OR = 2.37). This association was mirrored to a lesser extent for obesity and chronic hypertension (OR = 2.11).

Diabetes mellitus was positively associated with chronic hypertension (OR = 2.38) and associated to gestational hypertension (OR = 0.31). Hypothyroidism and dyslipidemia were positively associated with chronic hypertension (OR = 2.23 and 2.18, respectively).

As for ethnicity, being of Sub-Saharan Africa origin was associated with chronic hypertension (OR = 3.36) and pre-eclampsia (OR = 2.45). Finally, Maghreb origin was negatively associated with chronic hypertension (OR = 0.57).

## Discussion

We analyzed 17,177 pregnant women, 80.4% of the population originally included in the study. The prevalence of hypertension was 3.10%, which is like what reported in other studies, for example 3.6% in a population of Western Europe in 2019 [11]. Other studies found higher figures, such as 5.9% in Ireland in 2016 [12], and 7.4% in France in 2010–2018 [13]. Chronic hypertension accounted for 1.53% of all hypertensive women in our study, similarly to the 1.8% found by Olié et al. in France between 2010 and 2018 [13], and gestational hypertension accounted for 0.65%. The prevalence of pre-eclampsia was 0.78%, below the one found in Sweden (2.9%) and China (2.3%) in 2021 [14]. Differences in the prevalence of hypertension across populations May be attributed to variations in maternal age, the presence of obesity, or metabolic syndrome, all of which are associated with an increased risk [11]. Of the hypertensive disorders in pregnancy, pre-eclampsia is a common complication occurring in 2–5% of all pregnancies and is a major cause of maternal and neonatal morbidity and mortality [6].

The mean age of pregnant women with chronic hypertension, gestational hypertension or pre-eclampsia was 33.9 (± 6.2) years, 32.7 (± 5.5) and 30.6 (± 5.5) years, respectively. The mean age of pregnant women in our study is similar to the one observed in the cohort study conducted in the United States between 2009 and 2014, 30.1 (± 5.8) years [15].

In hypertensive women, the mean BMI [15] was 29.0 (± 6.3), while in pregnant women with pre-eclampsia it was 26.8 (± 5.8). In the rest of the pregnant women, it was 24.8 (± 4.9) [16]. Consistently, a retrospective cohort study in southern Spain [17] concluded that overweight and obesity have been associated with an increased risk of hypertensive disorders during pregnancy, and the risk was significantly higher as BMI increased. Moreover, in several population-based studies [11,18], Obesity has been associated with a two- to four-fold higher likelihood of developing pre-eclampsia.

The prevalence of diabetes mellitus in pregnant women with hypertension and, to a lesser extent, pre-eclampsia was increased in this study. Similarly, 70% increased risk of type 2 diabetes mellitus has been reported in pregnant women who had had hypertension in their first trimester [19]. Also, it was reported that hyperglycemia has been linked to impaired placental vasculogenesis, angiogenesis, and microvascular remodeling, which may be associated with an increased risk of pre-eclampsia [20,21]. Finally, diabetes mellitus and hypertension share risk factors that may further explain their association: age [22,23], overweight, and obesity [24–27]. In the Hyperglycemia and Adverse Pregnancy Outcome Study, pregnant women with poor cardiovascular health and an average gestational age of 28 weeks were reported to have a more than nine-fold higher prevalence of pre-eclampsia than those with ideal cardiovascular health [28].

We found the highest prevalence of hypothyroidism in pregnant women with chronic hypertension (12.5%). Consistently, some studies have established an association between abnormalities in the tests for thyroid function, such as subclinical hypothyroidism, and hypertension in pregnancy (OR = 1.6–3.4) [29–31]. However, other studies did not find this association [30,32]. Hypothyroidism during pregnancy has also been associated with an increased risk of pre-eclampsia [33].

In our study we have not observed an association between dyslipidemia and hypertension in pregnancy; however, the highest percentage of patients with dyslipidemia occurs in the case of preeclampsia; it is possible that a larger sample could demonstrate differences. Sharami et al observed that in the subjects with preeclampsia, serum triglyceride and total cholesterol levels were significantly increased and plasma HDL-cholesterol concentrations were decreased compared with the controls, but plasma LDL cholesterol levels didn’t differ between the two groups [34].

In the analysis of ethnic groups, 5.26% of pregnant Sub-Saharan African women had chronic hypertension and 2.03% had pre-eclampsia. Similarly, in a study in the US, a higher positive race/ethnicity association with chronic hypertension and pregnancy-induced hypertension was found in black women of Sub-Saharan African descent [23]. Moreover, different studies reported higher prevalence of pre-eclampsia among women of sub-Saharan African origin than among native white populations in Finland (3.0%, adjusted RR 1.8), France (Severe Pre-Eclampsia 1.6%, adjusted OR 2.5), and Australia, Canada, Spain, USA, Denmark, and Sweden (2.8%, adjusted OR 1.7) [35–37]. Finally, both black and South Asian women have been reported to have an increased risk of pre-eclampsia, after adjustment for confounding factors, in comparison with white women [38]. Similarly to the Finnish study [37], we found a lower incidence of gestational hypertension among Sub-Saharan African women. Moreover, the lower prevalence of gestational hypertension that we found among Maghreb women agrees with the findings from Bastola et al. [39]. This study also showed a significantly lower prevalence of hypertensive disorders among Middle Eastern and North African women in Finland, in comparison with Finnish women.

## Strengths and limitations

The first strength of the study lies in representativeness of sample, encompassing a significant portion of the population under investigation. Additionally, the implementation of universal screening protocols in pregnant women ensures comprehensive data collection, providing insights into the majority of participants and thereby enhancing the study’s representativeness and generalizability. Some of the potential limitations of the study include the racial-ethnic classification of women into just six broad categories. The use of broad ethnic classifications may obscure relevant intra-group heterogeneity in hypertension risk. Additionally, key social determinants of health (e.g., income, education, housing, prenatal care access) were not accounted for, limiting causal inference and contextual understanding. It is possible that there are variations among different subgroups within each category. Moreover, we did not analyse the socio-economic aspects as health determinants which may have influenced our results. It is conceivable that a subset of pregnant women sought prenatal care in facilities not affiliated with Social Insurance. This subgroup is estimated to constitute approximately 2.2% of the total population within our health region. However, owing to the comprehensive coverage provided by the Spanish National Health System, it is improbable that any missing data significantly impacted the study’s findings. Approximately 20% of the initial cohort was excluded due to missing data, and no comparison was made between included and excluded groups, limiting assessment of potential selection bias and sample representativeness. Among the limitations, it is important to note that the number of cases for certain hypertension subtypes may not be sufficient to establish associations with some complications due to sample size constraints. Another limitation arises from the retrospective nature of the study design, which may introduce biases in data collection. Furthermore, regarding the diagnostic criteria for hypertension, the study relied on ICD-10 classifications during the study period. Currently, the ISSHP 2021 guidelines are recommended [7], which could provide different insights.

## Conclusions

All subtypes of hypertension were associated with being overweight/obesity. Moreover, chronic hypertension was associated with diabetes and hypothyroidism. Regarding ethnicity, patients of Sub-Saharan African origin showed the highest prevalence of chronic hypertension and pre-eclampsia, patients from Asia/Middle East were at highest risk of gestational hypertension, and patients from the Maghreb had the lowest risk of chronic hypertension.

The study of hypertension subtypes in pregnancy provides a more precise epidemiological analysis of the distinct characteristics within different groups. Additionally, clinical management requires a multidisciplinary healthcare team to effectively address the disease burden. The association of various hypertension types with specific comorbidities, vascular risk factors, and ethnicity demonstrates that coordination between primary and secondary healthcare teams is essential to ensure optimal health management before, during, and after pregnancy [40]. Furthermore, hypertension subtypes can inform personalized prenatal care strategies, enabling early screening and targeted interventions for populations at elevated risk.

Population-based studies analyzing racial-ethnic differences in different subtypes of hypertension during pregnancy are needed for pathogenetic and epidemiological understanding and the management of hypertension during pregnancy.

### Informed consent

The databases from which the data were obtained are based on opt-out presumed consent and data are anonymized. If a patient decides to opt out, their data is excluded from the database. The need for Informed consent was waived by the Clinical Research Ethics Committee (CREC) of the Institut de Recerca IDIAP Jordi Gol.

### Human and animal rights

All procedures followed were in accordance with the ethical standards of the committee responsible for human experimentation (institutional and national) and with the Helsinki Declaration of 1975, as revised in 2008.
